# Cyber-bioethics: the new ethical discipline for digital health

**DOI:** 10.3389/fdgth.2024.1523180

**Published:** 2025-01-07

**Authors:** Robert Panadés, Oriol Yuguero

**Affiliations:** ^1^ Intelligence for Primary Care Research Group, Fundació Institut Universitari per a la recerca a l'Atenció Primària de Salut Jordi Gol i Gurina, Manresa, Spain; ^2^ Equip d'Atenció Primària d'Anoia Rural, Gerència d'Atenció Primària i a la comunitat Penedès, Institut Català de la Salut, Barcelona, Spain; ^3^ Digital Health Group, Family Medicine Society of Catalunya (CAMFIC), Barcelona, Spain; ^4^ E-RLab, Health Center, Univeritat Oberta de Catalunya (UOC), Barcelona, Spain

**Keywords:** ethics, artificial intelligence, cyber technology and ethics, autonomy, justice & inequalities

## Introduction

Bioethics, a term coined by Van Rensselaer Potter in his 1970 work “Bioethics, the Science of Survival”, is conceived as a bridge between “bios” and “ethos”. The word “bios” encompasses the totality of life, both somatic and rational, and represents biological knowledge. Meanwhile, “ethos” alludes both to the environment and to ethics, integrating knowledge of the human values which enable us to survive the challenges threatening environment survival. Potter aspired to a “global ethics” based on this knowledge, and saw bioethics as a newly emerging discipline which would forge a link between science and the humanities, or to be more exact, a bridge between the biological sciences and ethics. Bioethics was also a bridge to a peaceful, ecologically sustainable and socially equitable future (1). In 1971, the Joseph and Rose Kennedy Institute for the Study of Human Reproduction and Bioethics popularised this term (2).

While Potter saw bioethics as a new discipline, the philosophers and theologians of Georgetown considered it a branch of applied ethics. However, the events of the 1960s and 70s, such as the revelations in the press of experiments on uninformed patients and the civil rights and feminist movements, among other political, social and economic events, paved the way for the empowerment of “bioethicists” to advise on the ethical limits of medicine and biotechnology (3). At this time bioethics acquired the philosophical basis of principlism, which seeks to resolve bioethical problems with four principles, derived from the prima facie duties theory of WD Ross, as described in “The Principles of Biomedical Ethics” by Tom Beauchamp and James Childress: the principles of respect for autonomy, beneficence, non-maleficence, and justice (2, 4).

The Encyclopedia of Bioethics defines bioethics as the moral analysis of life and health. Applied ethics, which is interdisciplinary, encompasses such diverse spheres as medical bioethics, business ethics, and information ethics, among others. Potter highlighted the need to control technology and the importance of interdisciplinary collaboration (2).

Technologies like Artificial Intelligence (AI), mobile applications, wearables, and the Internet of Things are becoming integrated in what we know as Digital Health. Digital health promises countless benefits, but also throws up significant ethical challenges (5) which will affect how we live and coexist with others in the world (6), such as the dilemma of cybersecurity, data privacy (5), and the transformation of the doctor-patient-machine relationship.

Society stands at the crossroads of adapting bioethics to the 21st century in order to avoid the mistakes of the past. By adopting an ethical approach, society can move forward to a digital future which more equitable, more secure, and centred on the well-being of individuals (5). As in the 1960s and 70s, we believe that the rapid evolution in which we are immersed in the digital will bring with it the empowerment of a new discipline within bioethics. We know the biotechnology which Potter described and the ethics of AI, but we do not have a name to define the new bioethical discipline we propose, this new branch of bioethics in the digital era (5).

As well as digital health, AI is also receiving more attention, making it even more urgent and important to rise to the ethical and social challenges posed by AI. This has generated an avalanche of initiatives and documents relating to policies for action which not only identify ethical problems in AI, but also seek to provide guidance for such policies. In fact, many actors have proposed policies responding to AI from an ethical perspective, including governments and public bodies such as national ethics committees; technology companies like Google; engineers and their professional organisations such as the IEEE (Institute of Electrical and Electronics Engineers); intergovernmental organisations like the EU, with its Artificial Intelligence Regulation, the preamble to which was published on 13 March 2024, and will soon come into force (7); and non-profits, non-governmental bodies and researchers (6).

## Discussion

We propose the term “cyber-bioethics” to define this new discipline of bioethics which encompasses the new dilemmas of digital health. The word “cyber-bioethics” merges the cyber element and the word bioethics. Cyber is a prefix essentially deriving from cybernetics, and its use increased exponentially in the internet boom of the early 1990s (8).

“Cyber-bioethics” must be a flexible form of bioethics that adapts to new challenges; for example, if an artificial super-intelligence emerges which could acquire moral status, this would mean changes in our concepts of morality and responsibility (think of how we treat and perceive animals). It will need to be connected to a more general ethics of information and communication technologies, computer ethics, etc. (6).

In the field of cyber-bioethics, the classical principles of bioethics remain fundamental, but they need to be expanded and contextualised to address the challenges posed by the digitalisation of health, such as new principles related to data protection, cybersecurity, transparency, explainability, equity, non-discrimination, accountability, sustainability, as well as Asimov's Laws of robotics. To better define the principles, we propose that this branch of bioethics should engage with experts in ethics, law, medicine, computer science and engineering to address the complex challenges posed by the connection between technology and biology.

Patient autonomy has always been considered one of the basic principles of medical ethics. It emphasises the patient's right to make decisions about his or her own medical care, including treatment options, medical procedures and control over personal health information. The preservation of patient autonomy in the face of advancing AI technologies is crucial, as it directly influences the ability of individuals to make informed decisions about their medical care (9).

The main ethical challenge of AI in medicine relates to the privacy and confidentiality of patient data. The use of AI in this field involves the collection and analysis of large volumes of medical data, which raises concerns about the protection of personal information. Establishing robust regulations to protect this data and ensuring that patients' informed consent is used to authorise access and use of their information by healthcare staff is crucial (10), which is not without complexity in AI in healthcare, as it requires clear communication with patients about its use, especially with AI algorithms that can be difficult for non-experts to understand.

This includes detailing the implications of data sharing, the potential benefits and risks associated with AI-driven healthcare, and the level of human oversight in AI decisions (11).

Beneficence urges physicians to maximise the benefits for the patient. Therefore, when applying AI-based systems, physicians are expected to use the tools in a way that promotes the optimal outcome for the respective patient. Non-maleficence states that physicians have a fundamental duty not to harm their patients, either intentionally or through excessive or inappropriate use of medical means (12).

It is essential that AI developers work together with healthcare professionals to ensure that systems are transparent, explainable and free of bias (13).

With regard to beneficence and non-maleficence, explainability is a necessary characteristic of clinically applied AI systems. Explainability can be understood as a characteristic of an AI-driven system that allows a person to reconstruct why a given AI arrived at the predictions presented (12, 13).

Another key issue in the ethics of clinical AI is algorithmic bias, which can arise due to flaws in the design of scientific research or clinical trial studies, or in the data used to train the algorithms, and can result in improper or inappropriate medical decisions (14). It is critical to ensure that the algorithms used in AI are fair and free of these biases.

The principle of justice postulates that people should have equal access to the benefits of medical progress without ethically unjustified discrimination against any particular individual or social group (15).

The absence of adequate planning in the digital transformation can lead to the emergence of the digital divide, which refers to inequalities in access, use and skills in relation to Information and Communication Technologies (ICT) between different groups of people (16). To address problems such as the widening inequalities caused by the digital divide, digital determinants of health (DDS) should be taken into account. Like classical determinants, DDS can lead to differences in the health of individuals and communities. Digital literacy and the digital divide should therefore be included as additional axes of inequality, along with social class, gender, age, ethnicity and territory (16).

Within a framework of ethical and reliable AI in medicine, it is imperative to ensure the principles of equality, non-discrimination and solidarity, including the rights of people at risk of exclusion, in particular by paying special attention to situations affecting the most vulnerable groups, such as children, people with disabilities and others who have been historically disadvantaged or at risk of exclusion, as well as situations characterised by asymmetries of power or information, such as those that may occur between employers and employees or between companies and consumers (13).

With regard to the bias that AI in health may introduce, responsibility and accountability arise in the event of an erroneous decision. It is vital that measures are taken to ensure accountability in case of errors caused by AI, and to clearly define who is responsible in situations of harm or damage (10).

“Cyber-bioethics” touches on six main categories, including the ethics of machine training, the ethics of machine precision, patient-related ethics, physician-related ethics, shared ethics, and the roles of regulators (17), each of which touches on certain ethical issues as defined in the Table 1.

**Table 1:** Categories, subcategories of biocyberethics and related ethical issues.

Main categories biocybere	Subcategories	Ethical Issues
Ethics of machine training	Ownership of data	Proprietary rights of patie
		Ownership rights of health care providers, physicians, private or publicly owned hospitals
		Ownership rights of AI companies, designers, developers and manufacturers. Intellectual property
	Data Protection	• Confidentiality and patient privacy rights • Avoid both overprotection and underprotection. • Contextual rules must be clear
		• Cybersecurity • Responsibility of regulatory bodies overseeing the safety, efficacy and quality of medicines and medical devices at national and international level in each country or state. • Laws such as HIPAA help to formulate rules and train medical and technological personnel.
	Data exchange	Data sharing
Ethics of machine accuracy	Machine accuracy	• Efficiency, reliability or consistency in clinical tasks • Transparency inversely related to accuracy
	Transparency and intelligibility	Transparency in data selection, processing and decision making
	Black box phenomenon	
	Biases	
Patient-related ethics	Informed consent	
	Patient confidentiality Equality, non-discrimination and solidarity	
Physician-related ethics	Dependence and over-reliance on machines	
	Rivalry with machines and labour substitution	
	Trust	
	Empathy	
Shared ethics	Responsibility and culpability	In case of detrimental effects of AI use in medicine
	Responsibility	Responsibility of doctors in dealing with AI systems, even before harm occurs
	Costs	osts associated with the implementation of AI in medicine may be higher than expected cost savings
Role of regulators	Standardisation	
	Quality assurance	
	Marketing approval	Marketing approval
		CE Marking
	Licensing and ethical standard	Licensing and ethical standard
		HIPAA

HIPAA, Health Insurance Portability and Accountability Act; FDA, Food and Drug Administration; CE, Conformité Européenne; GDPR, General Data Protection Regulation.

In addition to the principles discussed above, the principles of cyber-bioethics should be aligned with ethical principles established by international bodies, such as the ethical principles of AI proposed by UNESCO in its Recommendation on the ethics of AI (2021) (18), which includes a series of general principles, in line with those previously presented by other international bodies such as the Future of Life Institute's Asilomar Principles (2017), the University of Montreal's Declaration for Responsible AI Development (2018) or the European Commission's Ethical Guidelines for Reliable AI (2019).

## Conclusion

“Cyber-Bioethics” represents an ethical response to the challenges posed by digital health and AI in healthcare. Its development must go hand in hand with the creation of robust regulatory frameworks, the incorporation of ethical principles in academic education and the implementation of inclusive and responsible technologies. Furthermore, “cyber-bioethics” must be dynamic and constantly adapt to evolving technologies, ensuring that digital health is used to improve healthcare in an equitable and responsible manner.

In the words of Potter (1), this would be a new discipline that would forge the union between current bioethics and biotechnology applied to digital health.

The future of digital health depends on an appropriate ethical integration of technologies into medical practice. It is essential that the principles of cyber-bioethics are adopted and promoted by all stakeholders, from AI developers to healthcare professionals and policy makers, to create a fairer, safer and more transparent digital health system.

We encourage their adoption and a broad discussion within the Bioethics community to address the moral challenges of rapidly evolving biotechnology.
